# At the Crossroads of Conspicuous and Concealable: What Race Categories Communicate about Sexual Orientation

**DOI:** 10.1371/journal.pone.0018025

**Published:** 2011-03-31

**Authors:** Kerri L. Johnson, Negin Ghavami

**Affiliations:** Departments of Communication Studies and Psychology, University of California Los Angeles, Los Angeles, California, United States of America; University College London, United Kingdom

## Abstract

We found that judgments of a perceptually ambiguous social category, sexual orientation, varied as a function of a perceptually obvious social category, race. Sexual orientation judgments tend to exploit a heuristic of gender inversion that often promotes accuracy. We predicted that an orthogonal social category that is itself gendered, race, would impact both sexual orientation categorizations and their accuracy. Importantly, overlaps in both the phenotypes and stereotypes associated with specific race and sex categories (e.g., the categories Black and Men and the categories Asian and Women) lead race categories to be decidedly gendered. Therefore, we reasoned that race categories would bias judgments of sexual orientation and their accuracy because of the inherent gendered nature. Indeed, both gay and straight perceivers in the United States were more likely to judge targets to be gay when target race was associated with gender-atypical stereotypes or phenotypes (e.g., Asian Men). Perceivers were also most accurate when judging the sexual orientation of the most strongly gender-stereotyped groups (i.e., Asian Women and Black Men), but least accurate when judging the sexual orientation of counter-stereotypical groups (i.e., Asian men and Black Women). Signal detection analyses confirmed that this pattern of accuracy was achieved because of heightened sensitivity to cues in groups who more naturally conform to gendered stereotypes (Asian Women and Black Men). Implications for social perception are discussed.

## Introduction

Social categorization is understood to be an important and foundational aspect of social perception [1], [2], [3]. Categorization brings to mind knowledge structures, or stereotypes, that shape subsequent attitudes and direct interpersonal interactions [1], [4], [3], [5]–[11]. Many, if not most, of the social categories that observers tend to spontaneously decode about others are visually apparent to the perceiver. These include sex, race, and age – categories that are perceived with a high degree of accuracy, in part because of these identities are strongly encoded in face and body cues [12]–[15]. Other social categories are arguably less obviously encoded in visual cues, but they may nevertheless be perceived with a surprising degree of accuracy.

It is now well established, for example, that other identities long described to be perceptually ambiguous [16]–[20] are inferred from visual cues with accuracy that exceeds chance. Visual information from faces and bodies elicits accurate sexual orientation judgments [21], [22]. Moreover, some of this work has suggested that perceptions of sexual orientation may occur spontaneously, based on minimal exposure to a visual stimulus [23]–[25]. Thus, it appears that perceptions of sexual orientation are both accurate and compulsory.

The accuracy in sexual orientation judgments appears to be facilitated from observers' exploitation of gender atypical cues. Indeed, gay men and lesbians tend to be more gender atypical, on average, than their straight counterparts. Specifically, such patterns are evident early in life [26]–[28], and they persist through adulthood [29]–[31]. Observers capitalize on these patterns by using a heuristic of gender inversion when making judgments of bodies [22], [32], [33], faces [34], and even descriptions about another person [35]. More often than not, applying a gender inversion heuristic to sexual orientation judgments promotes accuracy.

Scholars have frequently compared observers' ability to decode sexual orientation across a perceptually obvious social category – sex. Some notable effects have emerged. For instance, Ambady and colleagues [21] found that judgments of female, but not male targets were accurate for still images depicting the body. This finding was later elucidated by other research [22] in which gender-atypical body *motion* impacted sexual orientation judgments of both men and women, but gender-atypical body *shape* impacted only judgments of women. Such differences notwithstanding, perceivers' ability to decode sexual orientation across sex categories appears to exploit the common mechanism of perceived gender inversion [30], [35]. How judgments compare across other perceptually obvious categories remains less clear, however, due to a paucity of research exploring such questions. Nevertheless, judgments that occur when identities intersect have far-reaching implications not only for social categorization, but also the application of stereotypes and prejudice that occur thereafter.

Interestingly, a consensus is emerging in sex categorization research that implicates another perceptually obvious category – race – in the perception of sexual orientation. Specifically, race categories are decidedly gendered. Johnson and colleagues [36] found that sex category judgments varied systematically as a function of race. Black targets were associated with male-typed stereotypes and phenotypes; Asian targets were associated with female-typed stereotypes. This had implications for the efficiency of sex categorizations. Categorizations of men were more efficient for Black, relative to White or Asian targets, but categorizations of women were more efficient for Asian, relative to White or Black targets. Thus, overlaps in both stereotypes and phenotypes influenced sex categorizations in a gendered manner. Given the gendered nature of these percepts, we predicted that race category would also impact sexual orientation judgments.

Here we examined how race category impacts perceived sexual orientation, the accuracy of judgments, and the relative strength of the signal across sex and race categories. Participants judged the sexual orientation and gender-typicality of faces of men and women who were Black, White, and Asian. We predicted that accuracy would be highest for faces in which social categories were highly sex-typed, therefore making departures from the gender stereotype particularly likely to be noted by observers. Thus, we predicted that observers would be most accurate judging the sexual orientation of Black Men and Asian Women. These particular groups are likely to be perceived as highly gender-typical at the outset [36] therefore making deviations away from strong gendered expectations readily apparent. If correct, this should be evident not only in the overall accuracy of judgments, but also in higher sensitivity in signal detection analyses. Additionally, we predicted that observers would be least accurate when judging the sexual orientation of Black Women and Asian Men because these categories more naturally defy gendered expectations, and as a matter of course, observers would be prone to apply a heuristic of gender inversion, leading judgments astray, resulting in lower sensitivity in signal detection analyses. Finally, we tested these predictions in two populations – a sample of heterosexual undergraduates and a sample of self-identified gay men and lesbians.

## Materials and Methods

### Ethics Statement

All methods were reviewed and approved by the Institutional Review Board at the University of California, Los Angeles. All participants provided written informed consent and were treated in accord with the standards set forth by the American Psychological Association.

### Participants

Two samples of participants included 51 self-identified heterosexual undergraduates (10 men, 38 women, 3 unreported) who participated for course credit and a community sample of self-identified gay men (*n* = 10) and lesbians (*n* = 10) who participated for $10. Participants were not recruited based on their race category, but a majority of our participants were Caucasian.

### Stimuli

Stimuli included 300 photographs that were collected from public postings on dating websites in the United States. All websites were non-fee based, and photos were freely available for public viewing. Stimuli varied by sex (male, female), race (Black, White, and Asian), and sexual orientation (gay/lesbian, straight), yielding 25 stimuli per category. All determinations of sex, race, and sexual orientation were based on self-labeling within the individual's profile. Therefore, it was unnecessary for the experimenters to use subjective protocols to categorize each target's social category memberships. All images were cropped to depict only the face and were standardized for size. All individuals were devoid of facial hair and accessories.

Importantly, this technique is commonly used in studies examining the perception of sexual orientation [24], [25], although it is not without drawbacks. Targets, for instance, may misrepresent their social category memberships or strategically select their photos in online postings. The validity of social identity claims is difficult to verify. That said, such problems characterize *any* methods in which targets report their sexual orientation, and is therefore true of all social perception studies. Moreover, any misrepresentations of sexual orientation are likely to create noise within our findings, therefore working *against* our hypotheses. In spite of these limitations, the technique of utilizing such photographs remains the most widely used and least invasive method in the extant literature. We therefore elected to follow common experimental practice in our own research, although we return to this issue when discussing our results.

### Procedure

Participants provided two sets of judgments. First, participants categorized each target's sexual orientation using computer keys that were labeled “gay” and “straight.” Because each depicted target self-identified to be either gay or straight, this was the most reasonable measure of perceived sexual orientation. Each trial consisted of a fixation cross that appeared for 500 ms, followed by the face that appeared until the participant rendered a categorization. Within each block, photos were presented in random order using customized software. Participants received no feedback regarding the accuracy of their categorizations. Following this categorization task, participants also provided assessments of masculinity/femininity in a separate block of trials. For each photo, judgments were made using a 9 point scale anchored by *highly masculine* (−4) and *highly feminine* (4). Hereafter, we refer to this variable as Gender.

## Results

We sought to understand how race category impacted perceived sexual orientation, the accuracy of judgments, and the relative strength of the signal that conveyed sexual orientation across categories. Each question required a different analytic technique. Therefore, we first examined which factors determine sexual orientation judgments, irrespective of accuracy. Then we analyzed the accuracy of judgments two ways – one that compared the proportion correct across categories, and another that examined perceptual sensitivity (i.e., signal detection). Each approach provides unique information for the questions in our paper.

We used generalized estimating equations in all regression models because the outcomes (i.e., perceived sexual orientation and accuracy) were dichotomous and our design was within-subject [37]–[39]. We numerically coded and centered stimulus and participant characteristics along a common scale (Sex: male = −.5, female = .5; Race: Black = −.5, White = 0, Asian = .5; Sexual orientation: straight = −.5, gay = .5). This effect coding strategy is consistent with our other work (e.g., Johnson et al., 2010) in which race coding reflected the *a priori* ordering for perceived masculininity/femininity across race categories. We report unstandardized regression coefficients (*B*) and Wald *Z*s for each parameter.

### Preliminary Analysis of Perceived Gender

First we sought to replicate prior work [36] that supported the notion that race is gendered. We initially included participant sexual orientation (hereafter referred to as Population, to reflect recruitment) sample as a factor in this analysis. No effect involving Population reached significance; it was therefore dropped. We regressed perceived Gender onto target Race, target Sex, and the interaction (see Table 1). Relative to men, women were judged to be more feminine, *B* = 3.9168, *SE* = .1705, *z* = 22.97, *p*<.0001. Relative to Whites, Blacks were judged as less, but Asians as more feminine, *B = *.388, *SE = *.0416, *z* = 9.32, *p*<.0001. Although the interaction did reach significance, *B = *−.4757, *SE = *.0689, *z* = −6.91, *p*<.0001, the effect of race was common to both male and female targets, *B*s* = *.3129 and .0751, *SE*s* = *.0198, *z*s = 3.79, both *p*s<.001, differing only in the extent of the regression slope. Thus, these preliminary findings corroborate earlier work by establishing that the race of our targets was indeed perceived as gendered.

**Table 1:** Means for the percent of “gay” categorizations and proportion correct for sexual orientation judgments by race and sex.

	Target Race			
	Black	White	Asian	
Perceived Masulinity/Femininity				*Overall*
Male Targets	−2.27	−1.96	−1.64	−1.96
Female Targets	1.88	1.96	2.03	1.96
*Overall*	−0.19	.001	0.19	.001
Percent “Gay” Categorizations				*Overall*
Male Targets	30.72	37.02	43.79	37.02
Female Targets	26.59	26.52	26.45	26.52
*Overall*	28.62	31.54	34.61	31.54

### Perceived Sexual Orientation

Next, we examined how perceived sexual orientation varied as a function of stimulus characteristics and Population. Because perceivers tend to use gender atypicality as a cue for judging sexual orientation, we predicted that race category would impact judgments of sexual orientation such that the two groups for which race leads to perceived gender atypicality – Asian men and Black women – would be more likely to receive a gay/lesbian categorization.

We regressed perceived sexual orientation onto target Race, target Sex, sample Population, and all interactions (see Table 1). The effects of Sex, Race, and Population reached significance. Relative to men, women were 39% less likely to be categorized as gay, *B* = −.4867, *SE* = .0709, *z* = 2.42, *p* = .015, Odds Ratio (OR) = .6147. Relative to Whites, Blacks were 32% less likely, and Asians were 32% more likely to be categorized as gay, *B = *.2781, *SE = *.0538, *z* = 5.16, *p*<.0001, OR = 1.3206. Finally, gay men and lesbians were 51% more likely than straight participants to categorize a target as gay, *B* = .3994, *SE* = .1642, *z* = 2.42, *p* = .015 (*M*s = 27.39% and 35.99%), OR = 1.49.

As predicted, the interaction between race and sex also reached significance *B* = −.5712, *SE* = .1249, *z* = −4.57, *p*<.0001. Among male targets, gay categorizations were 75% more likely for Asians, but 75% less likely for Blacks, relative to Whites, simple *B* = .5637, *SE* = .0959, *z* = 5.88, *p*<.0001, OR = 1.7572. For female targets, the simple effect of race was not significant, simple *B* = −.0075, *SE* = .0665, *z* = −.11, *p* = .91. No interactions involving Population reached significance, all *B*s<.175, *p*s>.21.

These findings provide partial support for the notion that race category would impact perceptions of sexual orientation. Specifically, the race category most strongly associated with femininity (Asian) was also most likely to elicit a “gay” categorization for male targets. This finding is consistent with other research demonstrating that perceivers use a heuristic of gender inversion for making sexual orientation categorizations [22], [34]. An analogous pattern was not obtained for judgments of female targets. This finding is consistent with prior work in which the effect of gender-atypicality for social judgments has been consistently stronger for male, relative to female targets [22], [40]–[44].

### Proportion Correct

Although the pattern of results for *perceptions* of sexual orientation was partially consistent with our predictions, we were most interested in how observers may exploit cues that promote accuracy in judgments. The utilization of gender atypicality to inform judgments of sexual orientation promotes accuracy, in general [22], [34]. This occurs, at least in part, because gay men and lesbians exhibit a number of cues that are gender-atypical [30]. We predicted, however, that this heuristic might lead perceivers to misjudge the sexual orientation of Black women and Asian men. Because the race categories for these groups are stereotypically gender atypical, we predicted that this would likely compel a greater number of false alarms than for other intersections of race and sex therefore compromising accuracy. In contrast, we predicted that judgments of targets for which race and sex categories were extremely gender typical – Black men and Asian women – would be more accurate because departures from those expectations would be particularly salient to perceivers.

**Figure 1:**
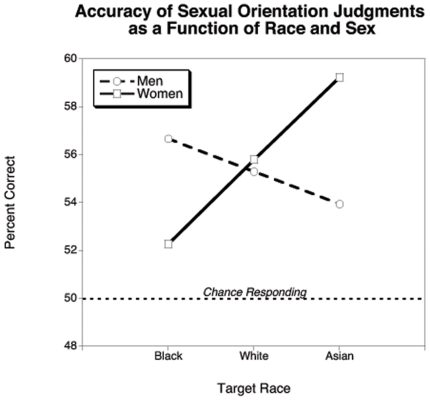
Percent correct sexual orientation categorizations as a function of target Race and target Sex. Chance responding is demarcated at 50%.

We coded accuracy numerically (0 = error; 1 = accurate) and regressed it onto Race, Sex, sample Population, and all interactions (see Figure 1). The effects of Race and Population reached significance. Relative to Whites, Blacks were 9% less likely, but Asians were 9% more likely to be accurately categorized, *B = *.0861, *SE  = *.0280, *z* = 3.08, *p* = .0021, OR = 1.0899. Gay men and lesbians' judgments were 7% more accurate than straight participants' judgments, *B* = .0665, *SE* = .0257, *z* = 2.59, *p* = .0096, OR = 1.0687 (*M*s = 54.72 and 56.37).

As predicted, the interaction between Race and Sex was also significant, *B* = .3930, *SE* = .08, *z* = 4.91, *p*<.0001. Among male targets, sexual orientation categorizations were 11% more accurate for Blacks, but 11% less accurate for Asians, relative to Whites, *B* = −.1104, *SE* = .0489, *z* = −2.26, *p* = .024, OR = .8955. For female targets, in contrast, categorizations were 33% more accurate for Asians, but 33% less accurate for Blacks, relative to Whites, *B* = .2826, *SE* = .0478, *z* = 5.81, *p*<.0001, OR = 1.3265. No interactions involving Population reached significance, all *B*s<.11, *p*s>.09.

Thus, overall accuracy was highest for the most highly sex-typed groups, Asian women and Black men, but lowest for counter sex-typed groups, Asian men and Black women. It may be that valid cues to sexual orientation – specifically those that are more likely be gender atypical in reality – are more easily detected in highly sex-typed targets. Theoretically, this may occur because the baseline expectations of these two groups are already extremely gendered making deviations from these extremes are more easily detected. If correct, this would be revealed in measures of sensitivity for judgments.

### Sensitivity

We also analyzed data using signal detection analyses that served two purposes. First, these analyses provided direct measures of sensitivity to cues that convey sexual orientation and to biases that are inherent in judgments. Second, although the global measures of accuracy reported above were informative, they were nevertheless incomplete insofar as they could not pinpoint the source of accuracy in perceptions (e.g., was accuracy high because of gay or straight categorizations?). Thus, the full accounting of accuracy *across* both gay and straight targets was warranted.

We computed sensitivity (*d'*) separately for each group using standard algorithms [45], see Table 2. To determine whether perceivers were sensitive to cues to sexual orientation at each intersection of sex and race, we computed separate one-sample t-tests that compared *d'* to 0. In every case, sensitivity was significantly above 0, *ts*(64) ranged from 3.73–13.23, all *p*s<.0001, Bonferroni corrected.

**Table 2:** Parameters and means for analyses of sensitivity (*d'*) and bias (*Beta*) in a signal detection analysis.

	Gay Targets		Straight Targets			
	Hits	Misses	C.R.	F.A.	*d'*	*Beta*
Men						
Black	.3363	.6637	.7757	.2243	.2514	1.3441
White	.4326	.5674	.6854	.3146	.2489	1.2200
Asian	.4365	.5635	.6168	.3831	.1135	1.0965
Women						
Black	.2568	.7432	.7988	.2012	.1840	1.5034
White	.3379	.6621	.7538	.2462	.2269	1.3484
Asian	.3097	.6903	.8586	.1414	.4920	2.2916

*Note*. Average rates of hits, misses, correct rejections (C.R.) and false alarms (F.A.) were collapsed across targets and participants.

Then we analyzed these data using a 2 (Sex)×3 (Race) repeated measures ANOVA. Population was initially included as a between subjects factor in this analysis. Neither the main effect nor any interaction involving population reached significance, so it was dropped from the analysis. Overall, sensitivity to cues for sexual orientation varied as a function of Sex, *F*(1, 67) = 10.74, *p* = .002 and Race, *F*(2, 134) = 3.331, *p* = .039. Importantly, these effects were qualified by a significant Race by Sex interaction, *F*(2, 134) = 29.513, *p*<.0001. Simple effects tests revealed that the effect of race was significant for judgments of both male and female targets, *F*s(2, 134) = 5.6 and 10.74, *p*s = .005 and<.0001, respectively. Among judgments of women, sensitivity was higher for Asian, relative to White or Black targets, *F*s(1, 67) = 28.21 and 42.00, *p*s<.0001, respectively. The opposite was true among judgments of men; sensitivity was lower for Asian, relative to White or Black targets, *F*s(1, 67) = 7.44 and 9.55, *p*s = .008 and .002, respectively.

To further probe these effects, we examined participants' threshold (i.e., *Beta*) for rendering a gay categorization using a 2 (Sex) by 3 (Race) repeated measures ANOVA. Interestingly, participants' criterion for making “gay” categorizations also varied as a function of Sex, *F*(1, 67) = 56.2812, *p*<.0001, Race, *F*(2, 134) = 30.2896, *p*<.0001, and their interaction, *F*(2, 134) = 24.0293, *p*s<.0001. Simple effects testing revealed that “gay” categorizations had a higher threshold for judgments of Black men, relative to Asian men, *F*(1,67) = 6.7852, *p* = .01; and a higher threshold for judgments of Asian women, relative to Black or White women, *F*s(1, 67) = 14.3527 and 26.8089, *p*s<.001.

These findings are consistent with the notion that Black men and Asian women are highly sex typed to begin with, thus corresponding to a bias to perceive these targets to be straight and requiring a high threshold for the signal to overcome initial stereotypes. Yet sensitivity was highest for these groups, suggesting that departures from gender typicality were readily apparent, and led to greater accuracy of judgments.

## Discussion

We found that judgments of a perceptually ambiguous social category, sexual orientation, varied as a function of a perceptually obvious social category, race. Sexual orientation judgments and their accuracy were consistent with findings that race is heavily gendered [36] thereby affecting categorizations through a heuristic of gender inversion [22], [34]. Indeed, this possibility was supported in preliminary analyses of perceived gender. Relative to Whites, Blacks were perceived to be masculine, but Asians were perceived to be feminine. This “race is gendered” pattern also had implications for the perception of sexual orientation. Perceivers were more likely to judge targets to be gay when the gendered race of a target was at odds with the target's sex (e.g., Asian Men). Finally, these patterns of judgments also impacted accuracy. Perceivers were most accurate when judging the sexual orientation of the most strongly gender stereotyped groups (i.e., Asian Women and Black Men). Perceivers' accuracy was achieved because of heightened sensitivity to valid signals.

Several aspects of this research warrant discussion. First, our use of images from dating websites, while standard practice in the field, may nevertheless affect our results in unmeasured, yet theoretically interesting ways. For example, the individuals who posted their photos on dating websites are likely to have strategically selected their profile images to suit a variety of motivations, and these motivations are likely to be inextricably tethered to the individual's social category-based identities. At times, intersecting identities may be at odds with one another. For instance, it may be that different race groups value gender-typicality to varying degrees. If correct, this could influence the selection of images to be posted online. Indeed, researchers are just beginning to understand the differences between racial and ethnic groups in both how they value gender typicality [46] and that these values may manifest in behaviors [47]. It is possible, therefore, that the most highly gender-typed groups (i.e., Black Men and Asian Women) are also the groups that strive most strongly to appear gender-typical. How these processes operate, in general, is largely speculative. Consequently, how such factors may be expressed among sexual minorities, specifically, is virtually uninvestigated. Ultimately, our research focused more on the *perception* of gendered cues and how they affect social categorizations among observers. As such, the questions that probe the *expression* of gender by different race groups is beyond the scope of the current manuscript. Nevertheless, we acknowledge that these factors are likely to have affected our targets' selection of photographs. We consider this issue to be an important domain for future investigation.

Additionally, the notion that social judgments may benefit from an in-group perceptual advantage has been documented in prior research, and it is important to consider within the context of the current findings. One form of in-group perceptual advantage that was addressed in our research is the tendency for gay perceivers to be more accurate in their judgments of sexual orientation, relative to their straight counterparts [21]. We also found a similar pattern. Overall, judgments made by our sample of gay men and lesbian perceivers were more accurate than judgments made by straight perceivers. It is noteworthy, however, that intersecting race and sex categories exerted the *same* impact for both populations. This suggests that the gendered nature of race categories affected perceptual judgments made by both gay and straight perceivers.

Another form of in-group perceptual advantage that we did not address in our research is the tendency for judgments of targets to be more accurate when they are a member of the perceiver's racial in-group. Specifically, the perception of in-group members compels more thorough visual processing [48], and this leads to heightened perceptual accuracy in some domains [49]. We did not recruit participants to test this possibility. It may be that perceiver race impacts the accuracy of sexual orientation judgments. Although some evidence suggests that there may *not* be an advantage for the accuracy of sexual orientation judgments made for same race targets [50], this remains an important avenue for future research.

Broadly speaking, these findings provide an important contribution to multiple literatures. First, these findings add to the burgeoning literature examining the perception of groups long thought to be perceptually ambiguous [51], [52]. Evidence is mounting that gendered visual cues are not only a valid indicator of sexual orientation [28], but also that the utilization of the cues promotes accuracy in social judgments [22], [34]. Our findings contribute to this growing body of research by highlighting the how gendered expectations of certain groups may facilitate or impair perceivers' ability to infer sexual orientation from gendered cues.

Second, these findings provide important information to the literature that examines the perception of intersecting social identities [36], [53], [54]. Specifically, these findings highlight the critical impact that orthogonal social categories can have on social perception. Moreover, they can help explain how evaluative judgments for particular groups, particularly Black women and Asian men, may be compromised due to apparent gender atypicality [22].

In sum, we have argued that the gendered nature of race categories carries implications for a social judgment that exploits gender typicality (or lack thereof) – sexual orientation. The findings provide important insights into social perception that occurs at the intersection of a range of social categories including sex, race, and sexual orientation. Moreover, these findings have direct bearing on our understanding of biases and evaluative judgments that occur at an intersectional group level.
